# An Initiative to Facilitate Park Usage, Discovery, and Physical Activity Among Children and Adolescents in Greenville County, South Carolina, 2014

**DOI:** 10.5888/pcd14.160043

**Published:** 2017-02-09

**Authors:** Melissa L. Fair, Andrew T. Kaczynski, S. Morgan Hughey, Gina M. Besenyi, Alicia R. Powers

**Affiliations:** 1 Arnold School of Public Health, University of South Carolina, Columbia, South Carolina; 2 LiveWell Greenville, Greenville, South Carolina; 3 Prevention Research Center, Arnold School of Public Health, University of South Carolina, Columbia, South Carolina; 4 Department of Clinical and Digital Health Sciences, College of Allied Health Sciences, Augusta University, Augusta, Georgia; 5 Furman University, Greenville, South Carolina

## Abstract

**Introduction:**

Parks are important settings for increasing population-level physical activity (PA). The objective of this study was to evaluate Park Hop, an incentivized scavenger-hunt–style intervention designed to influence park usage, discovery, park-based PA, and perceptions of parks among children and adolescents in Greenville County, South Carolina.

**Methods:**

We used 2 data collection methods: matched preintervention and postintervention parent-completed surveys and in-park observations during 4 days near the midpoint of the intervention. We used paired-samples *t* tests and logistic regression to analyze changes in park visitation, perceptions, and PA.

**Results:**

Children and adolescents visited an average of 12.1 (of 19) Park Hop parks, and discovered an average of 4.6 venues. In a subset of participants, from preintervention to postintervention, the mean number of park visits increased from 5.0 visits to 6.1 visits, the proportion of time engaged in PA during the most recent park visit increased from 77% to 87%, and parents reported more positive perceptions of the quality of park amenities. We observed more children and adolescents (n = 586) in the 2 intervention parks than in the 2 matched control parks (n = 305). However, the likelihood of children and adolescents engaging in moderate-to-vigorous PA was significantly greater in the control parks (74.3%) than in Park Hop parks (64.2%).

**Conclusion:**

Park Hop facilitated community-collaboration between park agencies and positively influenced park usage, park discovery, time engaged in PA during park visits, and perceptions of parks. This low-cost, replicable, and scalable model can be implemented across communities to facilitate youth and family-focused PA through parks.

## Introduction

In the United States, childhood obesity has become a priority public health issue, with more than one-third of the youth population overweight or obese (1). South Carolina has higher rates than the national average, and in Greenville County, South Carolina, 35.7% of young people are overweight or obese (2,3). Rates of obesity among young people are of concern given the increased risk of poor long-term health outcomes, including adult obesity, heart disease, diabetes, and some cancers (4,5). Physical activity (PA) is widely recognized as important to obesity prevention, and increased PA is correlated with reduced body mass index, improved physical fitness, and reduced risk of chronic disease (6). Despite these benefits, only about one-quarter of children or adolescents aged 6 to 15 participate in the daily recommended amount of 60 minutes of moderate-to-vigorous PA, with disparities found across sex, age, and racial/ethnic groups (6). Rates of PA significantly decrease during adolescence, making early intervention paramount to establishing life-long healthy PA habits (7).

Parks are important for increasing population-level PA because they are relatively inexpensive to operate and widely available (8,9). Parks, however, are often underused, and a significant number of observed park users are sedentary (10,11). Increased park discovery (defined as visiting a park for the first time) and awareness of parks can facilitate park usage and PA (12), but increasing park usage and discovery alone does not consistently increase levels of PA in parks (13). Organized programs and community outreach strategies may help increase PA levels in park settings (14), such as PA programs in parks and park structures (eg, playgrounds, walking trails) (15) and outreach strategies such as marketing and garnering community feedback (16).

Despite this evidence, few scalable park-based interventions to improve park usage, park discovery, perceptions of parks, and park-based PA targeting young people and families have been evaluated (17,18), and further research is needed to determine the efficacy of formal, organized park programs and outreach strategies (19). The objective of this study was to evaluate the 2014 Park Hop campaign in Greenville County, South Carolina. The goals of the evaluation were to determine whether an incentivized scavenger-hunt–style initiative in community parks would increase park usage, park discovery, perceptions of parks, and the proportion of time children and adolescents spend in PA during park visits. The study also sought to determine whether participation in the intervention influenced parents’ perceptions of park quality, quality of park amenities, park safety, and enjoyment of parks by their children.

## Methods

Park Hop took place in Greenville County, South Carolina, from mid-May to mid-August 2014. Greenville County has a population of 474,266 and comprises 69.5% white, 18.5% African American, 8.7% Latino, and 2.2% Asian residents (20). Young people (aged <18 y) make up 23.7% of the population (20). The annual median household income is $49,022, and 15.8% of residents live below the federal poverty level (20). The county has approximately 100 public park facilities operated by 7 parks and recreation entities.

LiveWell Greenville is a network of organizations working together to “make the healthy choice the easy choice” for Greenville County residents through policy, systems, and environmental change in several community settings. LiveWell Greenville’s At Play workgroup, composed of local parks and recreation organizations and other community partners, focuses on improving and supporting parks and trails systems to promote accessibility to safe, convenient places to be active. In 2013, Park Hop was created as a strategy to increase PA in parks.

**Figure 1:**
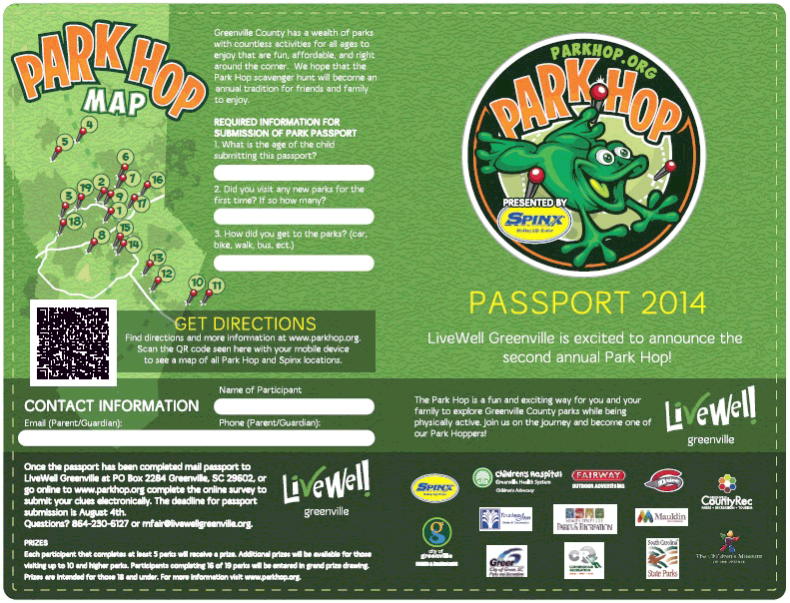
The Park Hop passport (front) used by children and adolescents to answer questions posed by clues placed in parks in Greenville County, South Carolina, 2014.

**Figure 2:**
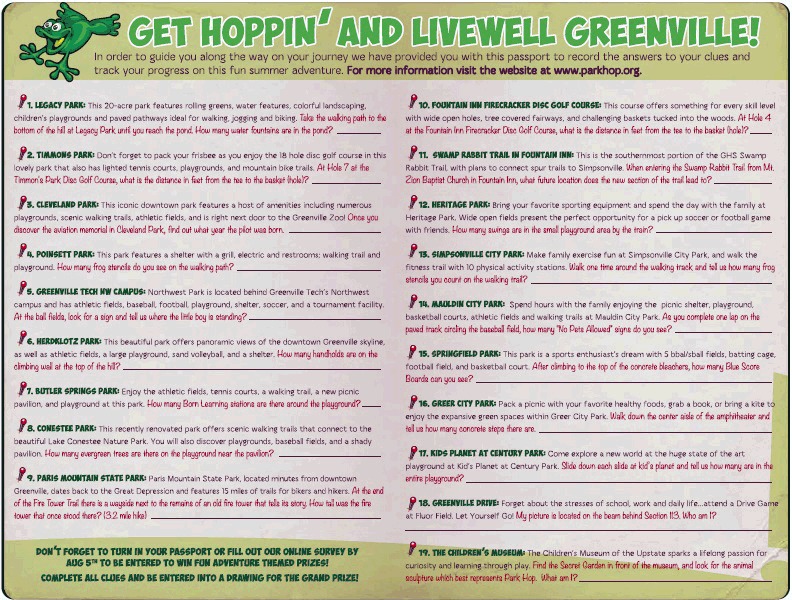
The Park Hop passport (back) used by children and adolescents to answer questions posed by clues placed in parks in Greenville County, South Carolina, 2014.

Park Hop is part of a community action plan developed by the At Play workgroup using recommendations from the Centers for Disease Control and Prevention’s Guide to Strategies to Increase Physical Activity in the Community (21). Park Hop is a summer-long scavenger hunt designed to increase park usage, park discovery, and the proportion of time spent in PA during park visits by incentivizing youth and their families to visit park venues in Greenville County (22). In 2014, 19 parks were included in the program. Each park was assigned 1 clue, and each clue focused on a park amenity designed for PA (eg, playground, rock climbing wall, walking trail) and prompted participants to be active in searching for the clue during their visit (eg, walk the trail and count the number of frog stencils). Participants answered the clues using a printable park passport (Figure 1 and Figure 2) or mobile application (app) for a chance to win adventure-themed prizes. Three prize levels were designated according to the number of clues answered (5–9, 10–15, and 16–19 clues); the value of prizes increased with each level. Participants could answer clues at any point during the intervention at their own pace.

**Figure 3:**
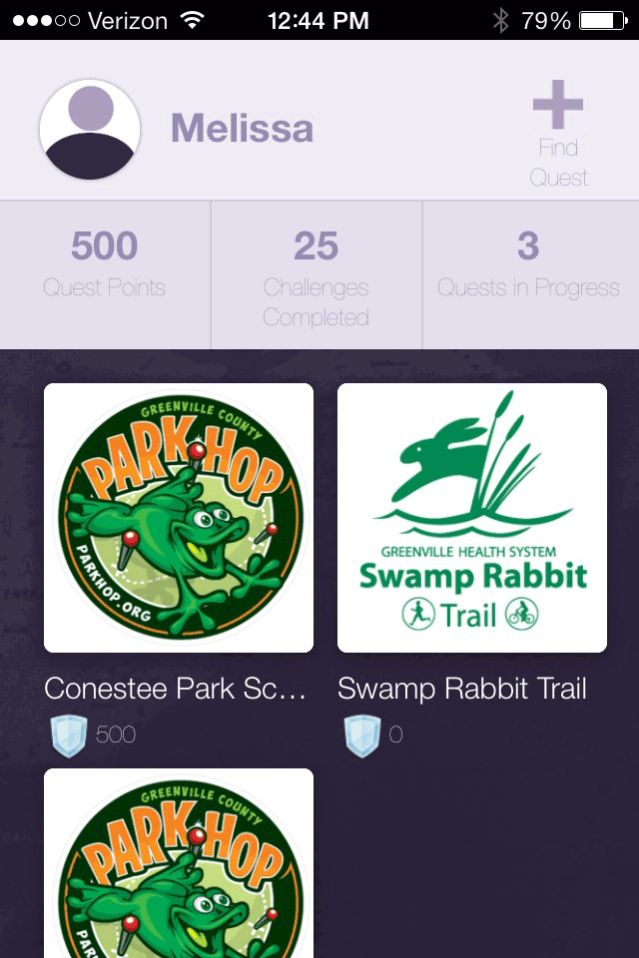
Screen shot of home screen, Park Hop mobile app, Greenville County, South Carolina, 2014.

**Figure 4:**
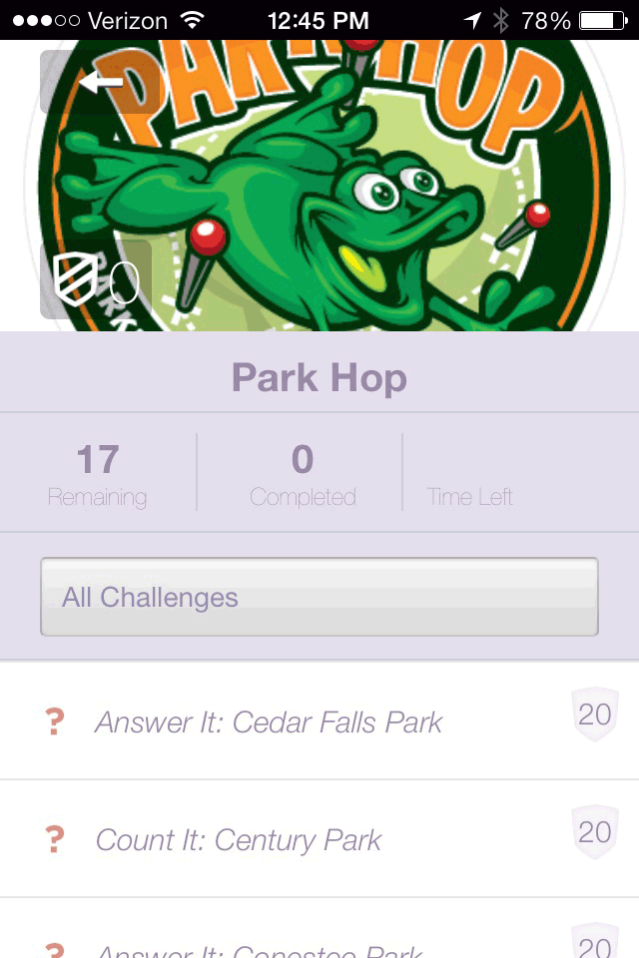
Screenshot of the list of parks included in the scavenger hunt, Park Hop mobile app, Greenville County, South Carolina, 2014.

**Figure 5:**
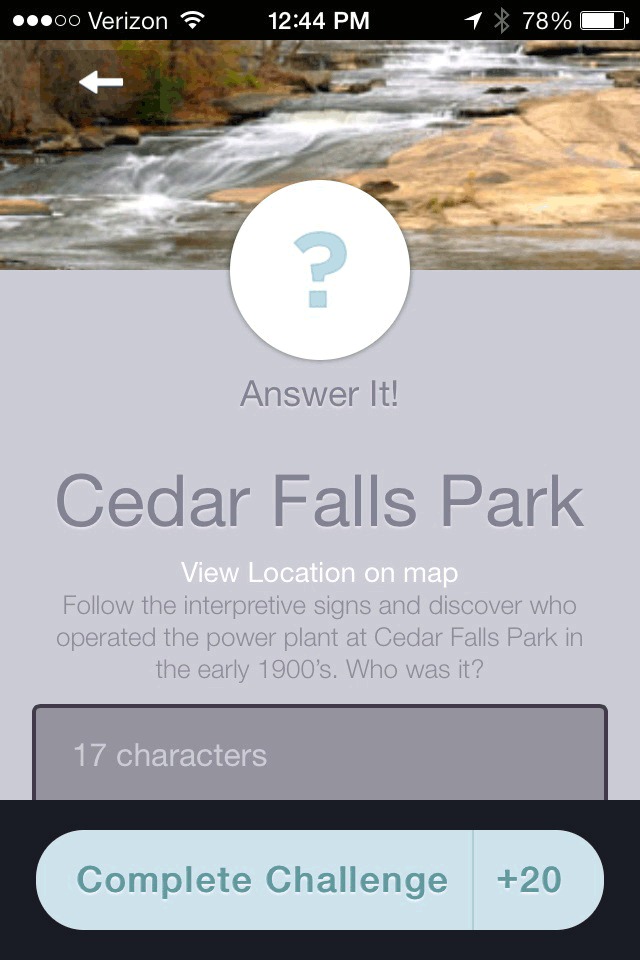
Screenshot showing an example of a Park Hop park clue, Park Hop mobile app, Greenville County, South Carolina, 2014.

Park Hop participation was open to all young people aged 18 years or younger and their families. Participants were recruited through advertising in multiple media outlets and through stickers and flyers distributed to elementary schools and local community centers. Participants registered online by visiting the Park Hop website, which provided access to the printable park passport, or by downloading the mobile app from the Apple or Android app store (Figure 3, Figure 4, Figure 5). Participants submitted their completed passports (ie, answers to clues) online or through the app.

### Measures

We used 2 methods of data collection to evaluate Park Hop: preintervention and postintervention surveys completed by parents and direct observations in parks. Surveys asked questions about participants’ demographic characteristics, park usage, park discovery, park-based PA, and perceptions of parks. The System for Observing Play and Recreation in Communities (SOPARC) assessed PA expenditure in a subset of 2 matched intervention parks and 2 control parks. The study was approved by the institutional review board at the University of South Carolina.

**Surveys.** We collected survey data from mid-May to mid-August 2014. Preintervention surveys were emailed to each registered participant’s parent or guardian within 1 week of registration and were available for 7 days. Postintervention surveys were emailed to parents or guardians of all registered participants who submitted a Park Hop passport online or through the mobile app after completion of the program and were available for 2 weeks. Only parents or guardians of active program participants were eligible to complete the postintervention survey. For pre- and postintervention comparisons of survey data, we matched participants using the date of birth for the child or adolescent and the email address of the parent or guardian. For parents or guardians with more than 1 participating child, we asked for information on the child with the birthday closest to January 1 of the calendar year. Participants who completed either survey had a chance to win a $25 Visa gift card.

Park usage was assessed by using 3 survey questions. One question in both the preintervention and postintervention survey asked about the number of park visits in the previous 30 days (23). A second question (in both surveys) asked about the total time (in minutes) of the most recent park visit. A third question, asked only in the postintervention survey, asked how many (and which) of the 19 intervention parks were visited as part of Park Hop. 

Park discovery was assessed through 1 postintervention survey question that asked how many (and which) of the 19 intervention parks were visited for the first time as part of Park Hop. Park-based PA was assessed at preintervention and postintervention by using questions from the Physical Activity in the Park Setting questionnaire (23). The first question asked about the total time (in minutes) of the most recent park visit and then the total time (in minutes) engaged in PA during the most recent park visit. Responses were used to calculate the proportion of time engaged in PA during park visits. PA was defined “as any physical movement other than sitting (eg, walking, biking).”

Parent or guardian perceptions of parks in Greenville County were assessed preintervention and postintervention by using 4 survey questions. Perception of park safety was measured with 1 item rated on a scale from 1 (very unsafe) to 5 (very safe). Perceptions of overall quality of parks and quality of park amenities were measured with 1 item for each rated on a scale of 1 (poor) to 5 (excellent). Perceived enjoyment of parks was measured with 1 item asking parents or guardians to rate on a scale of 1 (strongly disagree) to 5 (strongly agree) their agreement with the following statement: “My child enjoys parks in Greenville County.” We collected data on the demographic characteristics (age, sex, height, weight, race/ethnicity, income, and ZIP code) of participating children and adolescents in the postintervention survey. We used weight status categories for children and teenagers established by the Centers for Disease Control and Prevention (24) to categorize the body mass index of participants: underweight (<5th percentile), normal weight (≥5th to <85th percentiles), overweight (≥85th and <95th percentiles), and obese (≥95 percentile).

**Park observations.** Near the midpoint of the intervention, trained data collectors completed direct observations using SOPARC to assess PA levels of park users in 2 Park Hop parks and 2 non-Park Hop parks (25). Using existing geographic information systems (GIS), park audit, and census tract data, we matched Park Hop and control parks on size of park, park amenities, and demographic characteristics of the surrounding community. Using SOPARC protocols (25), we divided each park into observable spaces (target areas). Each data collector had a park map and moved through the target areas to conduct systematic momentary scans of park users. Each scan recorded the age group, sex, race/ethnicity, and PA intensity level (sedentary, walking, vigorous) of users (26). Research has established construct validity for the SOPARC activity intensity codes via accelerometers and heart monitors (25), and at least 2 studies reported high inter-rater reliability using these observation methods in parks (11,14).

Before observation, data collectors attended an 8-hour training session. This session included practice videos and real-time observations in parks until 100% inter-rater reliability was achieved for the number of observed users and their PA intensity level. A SOPARC observation schedule of 4 days per week and 4 times per day was selected, because studies showed that this schedule was sufficient for estimating park-based PA (27). Data were collected in all 4 parks during 4 simultaneous observation periods (9:30 AM, 11:30 AM, 3:30 PM, 6:00 PM) on 4 days (Thursday, Friday, Saturday, and Sunday).

### Analysis

Both survey and direct observation measures were analyzed using SPSS 22 (IBM Corp). We determined the number of parks newly discovered. Paired samples *t* tests were used to determine any significant changes in the number of park visits per month, time spent during park visits, proportion of time engaged in PA during park visits, and perceptions of parks. *P* values were significant at ≤.05. Logistic regression, controlling for age group, sex, and race/ethnicity, was used to examine the likelihood of children or adolescents observed engaging in moderate-to-vigorous PA in Park Hop and control parks.

## Results

A total of 746 families registered to participate, with an average of 2.2 youths per household for an estimated 1,678 children and adolescents. Completed park passports were submitted by 302 children and adolescents from 236 families through the online portal or mobile app, and another 211 children and adolescents visited at least 1 park while using the mobile app for a total of 513 active participants. Among those that completed the park hop passport, 28.9% of participants used the printable park passport, 46.1% used the mobile app, and 25% used both. 

The preintervention survey was completed by 278 (of 746) registered participants’ parents or guardians for a completion rate of 37.3%; 122 postintervention surveys were received from 236 families for a completion rate of 51.7%. Pre- and postintervention surveys were matched for 77 participants.

Postintervention survey participants ranged in age from 2 to 17 years (mean, 8.2 y, standard deviation [SD], 3.1 y). Most participants were boys (62.7%), white (90.3%), and non-Hispanic (95.9%). Annual household income ranged from $25,000 to more than $200,000, with most (64.2%) annual incomes greater than $50,000. Most participants (72.4%) were normal weight, 5.2% were underweight, 8.6% were overweight, and 13.8% were obese. 

According to the postintervention survey, the number of parks visited in the previous 30 days ranged from 1 to 19 (mean, 12.1; SD, 6.1). Of these, 15.9% of participants visited fewer than 4 parks, 19.7% visited 5 to 9 parks, 15.8% visited 10 to 15 parks, and 48.7% visited 16 to 19 parks. Among families that completed both the preintervention and postintervention survey, the mean number of parks visited during the previous 30 days increased significantly by 1.1 park (*t* = 1.97, *P* = .05) (Table). Park discovery ranged from 0 to 14 (mean, 4.6; SD, 3.7) parks visited for the first time, with all but 1 participant visiting at least 1 new park. The total time reported for park visits was not significantly different between preintervention (128 min) and postintervention (114 min) (*t* = −1.21, *P* = .23). The total time engaged in PA during the most recent park visit was also similar preintervention (98.6 min) and postintervention (99.2 min), but the proportion of time spent in PA during the most recent park visit increased significantly from preintervention (77%) to postintervention (87%) (*t* = 2.85, *P* = .006). 

**Table:** Park Visitation, Physical Activity, and Park Perceptions Among 77 Respondents to a Preintervention Survey and Postintervention Survey in Park Hop, Greenville County, South Carolina, 2014^a^

Variable	Mean (Standard Deviation)		Difference Between Preintervention and Postintervention	
	Pre	Post	*t*	*P* Value^b^
Park visits per month (previous 30 days)	5.0 (4.2)	6.1 (4.2)	1.97	.05
Time spent during last park visit (min)	128 (68.3)	114 (76.7)	−1.21	.23
Percentage of time spent in physical activity during last park visit	77 (26)	87 (17)	2.85	.006
Perception of safety of parks^c^	4.3 (0.5)	4.4 (0.6)	0.75	.45
Perception of quality of parks^d^	4.4 (0.8)	4.5 (0.6)	1.03	.31
Perception of quality of park amenities^d^	3.5 (0.9)	4.3 (0.6)	6.83	<.001
Perception of child enjoyment of parks^e^	4.8 (0.4)	4.9 (0.4)	−0.63	.53

a Respondents were the parents or guardians of children and adolescents participating in Park Hop. Of 746 registered participants, 278 completed the preintervention survey and 122 completed the postintervention survey. Pre- and postintervention surveys were matched for 77 participants by using a date of birth for the child or adolescent and the email address of the parent or guardian.

b All *P* values obtained by using independent-samples *t* tests.

c Measured with 1 item rated on a scale of 1 (very unsafe) to 5 (very safe).

d Measured with 1 item rated on a scale of 1 (poor) to 5 (excellent).

e Measured with 1 item asking parents or guardians to rate on a scale of 1 (strongly disagree) to 5 (strongly agree) their agreement with the following statement: “My child enjoys parks in Greenville County.”

Parent perceptions of park safety, overall quality of parks, or youth enjoyment of parks did not change significantly, but perceptions of the quality of park amenities improved significantly from 3.5 to 4.3 (*t* = 6.83, *P* < .001) (Table).

We observed 891 children and adolescents in the 4 matched parks: 586 participants in the intervention parks and 305 participants in the control parks. The likelihood of children and adolescents being observed engaging in moderate or vigorous PA was significantly greater (74.3%; odds ratio, 1.61; 95% confidence interval, 1.19–2.20) in the 2 control parks than in the 2 Park Hop parks (64.2%).

## Discussion

Our study indicates that Park Hop can facilitate park usage and park discovery, increase time engaged in PA during park visits, and enhance perceptions of parks. Similarly, a previous study demonstrated community-based park programming resulted in significant increases in park visits and frequency of exercise participation (16), while another study reported that added park programming and renovations to key park facilities (eg, playing fields) resulted in significant increases in park visitation by young people and adults (28). However, few park-based interventions have evaluated how park programs can influence changes in PA behaviors and perceptions (29).

Many interventions that are focused on the built environment, and particularly parks, emphasize environmental changes by renovating or adding amenities, which can be expensive and infeasible for many communities (29). In contrast, Park Hop maximizes use of existing park facilities and encourages park visitation. Our evaluation provides preliminary evidence that such programming may increase park usage and improve the PA behavior of children and adolescents.

Descriptions of similar interventions are sparse in the public health literature, but these interventions are highly recommended by experts in public health and parks and recreation (15,29). Innovative aspects of Park Hop include the technology used in the mobile application. To further promote PA, the mobile application will be developed to add such features such as “park near you” push notifications, extensive in-park scavenger hunts, and a database of year-round clues for each of the county’s 100 or so parks. Park Hop embodies a successful community collaboration working to cross-promote parks and recreation environments to increase active living in Greenville County (22). This collaboration of volunteer representatives from parks and recreation agencies, nonprofit organizations, and for-profit organizations was integral to program design, implementation, and evaluation.

A strength of this study was the use of direct observation methods (ie, SOPARC) to compare PA levels in a matched subset of Park Hop parks and control parks. Although we observed more children and adolescents in Park Hop parks, we observed a greater percentage of children and adolescents engaging in moderate-to-vigorous PA in the control parks. This finding differs from those of previous studies that used SOPARC to evaluate park interventions, particularly park renovations (16,28). The built environment and public health literature consists primarily of cross-sectional studies (30), so the use of pre- and postintervention survey measures contributes to better understanding the effects of Park Hop.

This study has several limitations. First, the number of matched pre- and postintervention surveys was small, and not all surveys could be matched. Therefore, our study participants may not be representative of the entire sample of Park Hop families. Second, both the pre- and postintervention surveys relied on self-reports from parents or guardians, which may have biased the results; however, the survey allowed us to expediently capture data on various constructs that are difficult to measure objectively (eg, park quality perceptions). Finally, although SOPARC measures provided an objective assessment of park-based PA, we could not be certain that the children and adolescents we observed were Park Hop participants. In future studies, individual-level PA (versus park-level or target area-level) measurement is needed to further elucidate the role of Park Hop in facilitating PA among participants, including a rigorous assessment of pre–post effects using GPS (global positioning system) and accelerometers linked to mobile app technology (29).

Park Hop is a valuable community-based initiative that encourages young people and their families to visit, discover, and be active in local parks during the summer. Using multiple types of evaluation measures, we found preliminary evidence that Park Hop increased park usage and discovery, helped facilitate PA during park visits, and increased positive perceptions of local parks. With further refinement and dissemination, sustainable community interventions such as Park Hop can play an important role in increasing active living among children and adolescents and their families (16,29) A future objective of Park Hop is to target underserved communities and settings, including out-of-school-time centers and church youth organizations who serve racial/ethnic minority and low-income populations. Additionally, the mobile app will be expanded to engage users and increase PA through wireless point-of-decision prompts located in parks. Lastly, Park Hop will be shared as a low-cost, replicable, and scalable model throughout the state and region.
